# SAM3-AgeSeg: an adaptive segmentation model for bone tumors in the aging population

**DOI:** 10.3389/fmed.2026.1939752

**Published:** 2026-08-17

**Authors:** Fang Zhang, Xiaoling Yuan, Hongxia Song, Yueying Chen, Tianting Bai

**Affiliations:** Department of Orthopedics, Jinling Hospital, Affiliated Hospital of Medical School Nanjing University, Nanjing, China

**Keywords:** adaptive learning, aging population, bone tumor, medical image segmentation, SAM3

## Abstract

**Background:**

Accurate segmentation of bone tumors from X-ray images is crucial for clinical diagnosis and treatment planning. However, elderly patients pose a significant challenge to general-purpose segmentation models.

**Methods:**

To address this aging-related medical challenge, we propose SAM3-AgeSeg, an adaptive segmentation model specifically designed for the aging population. Built on the powerful foundation model SAM3, our approach uses a lightweight fine-tuning technique, Low-Rank Adaptation (LoRA), to efficiently learn and adapt to the imaging characteristics of elderly bones.

**Results:**

We conducted a systematic evaluation of the public BTXRD bone tumor X-ray dataset. The experimental results demonstrate that SAM3-AgeSeg outperforms existing state-of-the-art methods in overall segmentation accuracy and exhibits superior boundary segmentation robustness, particularly for a subset of elderly patients.

**Discussion:**

This study validates the effectiveness of adaptive strategies in enhancing the performance of medical image analysis for aging-related challenges, offering a potential research direction for further investigation into age-adaptive medical image segmentation.

## 1 Introduction

Primary bone tumors, particularly malignant ones such as osteosarcoma, although relatively rare, pose a serious threat to patient health (1, 2). Early and accurate imaging diagnosis is crucial for improving patient prognosis and formulating personalized treatment plans. X-ray examination, due to its high accessibility and low cost, serves as the first-line imaging modality for bone tumor screening and preliminary diagnosis. However, bone tumors exhibit complex and diverse manifestations on X-ray images, and their benign vs. malignant differentiation as well as precise lesion boundary delineation heavily rely on the radiologist's experience, which can easily lead to missed diagnoses and misdiagnoses in clinical practice (3–5).

In recent years, artificial intelligence technologies, especially deep learning, have made significant progress in medical image analysis, bringing new opportunities for computer-aided diagnosis. Several research efforts have been devoted to tasks such as detecting, segmentating, and classifying bone tumors in X-ray images (6–10). However, existing methods face a severe challenge when addressing an increasingly prominent clinical reality: global population aging. With the acceleration of societal aging, the number of elderly bone tumor patients is continuously increasing. The imaging characteristics of elderly patients differ from those of younger populations, introducing additional complexity to X-ray image analysis (11, 12). This leads to a significant decline in the segmentation accuracy and robustness of general-purpose models trained on data from younger populations when applied to elderly patients. Currently, research on intelligent segmentation models specifically optimized for the imaging characteristics of bone tumors in the aging population remains largely unexplored.

To address this challenge, this study aims to develop an accurate bone tumor segmentation model that can adapt to the imaging features of the aging population. We note that visual foundation models have demonstrated powerful zero-shot and few-shot learning capabilities in general image segmentation tasks. Inspired by this, we propose the SAM3-AgeSeg model. The core idea of this model is not to train a large model from scratch, but to leverage the powerful visual foundation model SAM3 (13) and rapidly adapt it to the specific and challenging visual domain of “elderly bone tumors” through efficient fine-tuning strategies. Specifically, we introduce a parameter-efficient fine-tuning technique called Low-Rank Adaptation (LoRA), allowing the model to retain general visual knowledge while focusing on learning the tumor boundary features commonly observed in elderly patients. We conducted systematic validation of the proposed method on the currently largest publicly available bone tumor X-ray dataset, BTXRD (14). Experimental results show that, compared with existing state-of-the-art methods, SAM3-AgeSeg not only achieves superior segmentation performance on the overall test set but also demonstrates enhanced robustness in boundary segmentation for the subset of elderly patients.

The main contributions of this study can be summarized as follows:

- We propose SAM3-AgeSeg, an adaptive segmentation framework specifically designed for the aging population. This framework takes the visual foundation model SAM3 as its core and efficiently adapts to the imaging features of elderly bone tumors through lightweight, parameter-efficient fine-tuning strategies, avoiding the enormous computational overhead of training from scratch.
- We design an age-aware fine-tuning strategy tailored to the imaging characteristics of elderly bone tumors. By introducing LoRA modules, the model can accurately capture boundary features commonly observed in elderly patients while preserving general visual priors, thereby significantly improving segmentation accuracy for the elderly population.
- We conduct extensive experimental validation on the large-scale publicly available bone tumor X-ray dataset BTXRD. Through comparisons with existing state-of-the-art methods and specialized evaluations on the elderly patient subset, we demonstrate that SAM3-AgeSeg outperforms existing methods in both overall performance and robustness in aging scenarios, providing preliminary evidence supporting the potential of age-aware adaptation for elderly bone tumor segmentation.

## 2 Related works

### 2.1 Medical image segmentation

Fully supervised medical image segmentation has long been dominated by CNN-based encoder-decoder architectures, most notably the U-Net family (15–17), which preserve fine spatial details through skip connections and deliver stable performance across organ- and lesion-segmentation tasks. The subsequent introduction of Vision Transformers and hybrid CNN-Transformer designs further improved global contextual modeling, benefiting complex anatomical structures (18–20). Nevertheless, such specialist models are typically trained from scratch for specific modalities, anatomical regions, or pathologies, limiting their generalization across diverse clinical scenarios and requiring extensive manual annotations for each new task (21, 22). The emergence of large-scale vision foundation models, exemplified by the Segment Anything Model (SAM) (23), has shifted the paradigm toward generalist segmentation with strong zero-shot and interactive capabilities. Recent studies have attempted to adapt SAM to medical imaging through full fine-tuning, adapter layers, or parameter-efficient approaches such as LoRA (11, 24). However, most existing medical adaptations of SAM still rely primarily on geometric prompts (points or bounding boxes) and lack the ability to ground clinical semantic concepts such as bone tumor or osteolytic lesions. Moreover, their robustness deteriorates when applied to elderly patients. Addressing this vulnerability remains an open challenge.

### 2.2 Promptable segmentation foundation models

Interactive medical segmentation predates recent foundation models and commonly refines an automatic prediction with lightweight user inputs such as clicks or scribbles (25). SAM formalizes a promptable interface via a prompt encoder and mask decoder, enabling segmentation conditioned on spatial prompts (23), and SAM2 extends this design with a memory mechanism for streaming image and video settings (26). Direct application of the original SAM to medical data often reveals performance degradation due to domain shift. Medical adaptations such as MedSAM (24) and Medical SAM Adapter (27) address this through supervised domain adaptation or parameter-efficient customization. The more recent SAM3 (13) introduces promptable concept segmentation (PCS), which accepts open-vocabulary natural language descriptions as prompts in addition to geometric cues, offering a conceptually closer interaction paradigm to clinical practice. Works such as MedSAM3 (28) and Medical SAM3 (29) fine-tune SAM3 on large-scale, heterogeneous medical datasets to align textual medical concepts with anatomical targets, demonstrating that holistic domain adaptation improves both semantic grounding and segmentation reliability. However, none of these models have been specifically optimized for the imaging characteristics of the aging population, such as blurred lesion boundaries commonly observed in elderly patients or the scarcity of elderly samples in training data, nor do they incorporate age as a key covariate in model design. This motivates the present study, which aims to develop an age-aware adaptation of the SAM3 architecture for bone tumor segmentation in the elderly.

## 3 Method

### 3.1 Problem definition

Bone tumor segmentation aims to automatically identify and delineate the precise boundaries of bone tumors in patients' X-ray images. In this work, the input sample is a bone tumor X-ray image, which is represented as a pixel matrix containing imaging features of bone structures, tumor morphology, and surrounding soft tissues. These features encompass multi-dimensional information, including tumor size, shape, margin sharpness, density distribution, and age-associated bone changes such as reduced bone mineral density and cortical thinning. Formally, given a bone tumor X-ray image *X*, our objective is to predict the corresponding tumor segmentation mask *Y*. *Y* is a binary matrix of the same dimensions as *X*, with each pixel value indicating whether that location belongs to the tumor region. We formulate this segmentation problem as learning a non-linear mapping *Y* = *f*(*X*; Θ), where Θ represents the learnable parameters of the model. During the training phase, the model optimizes its parameters by minimizing the discrepancy between the predicted mask Ŷ and the ground-truth mask *Y*_gt_, typically using a combination of Dice loss and cross-entropy loss. Given that bone tumors in X-ray images often exhibit blurred boundaries, irregular shapes, and low contrast with surrounding normal bone tissue, the segmentation model must simultaneously capture global skeletal anatomical context, local tumor features, and age-associated imaging patterns. Blurred boundaries are particularly pronounced in elderly patients. For the elderly patient population, the model must also adapt to age-associated imaging characteristics of the bones to enable accurate tumor boundary localization.

### 3.2 Overall framework

**Figure 1:**
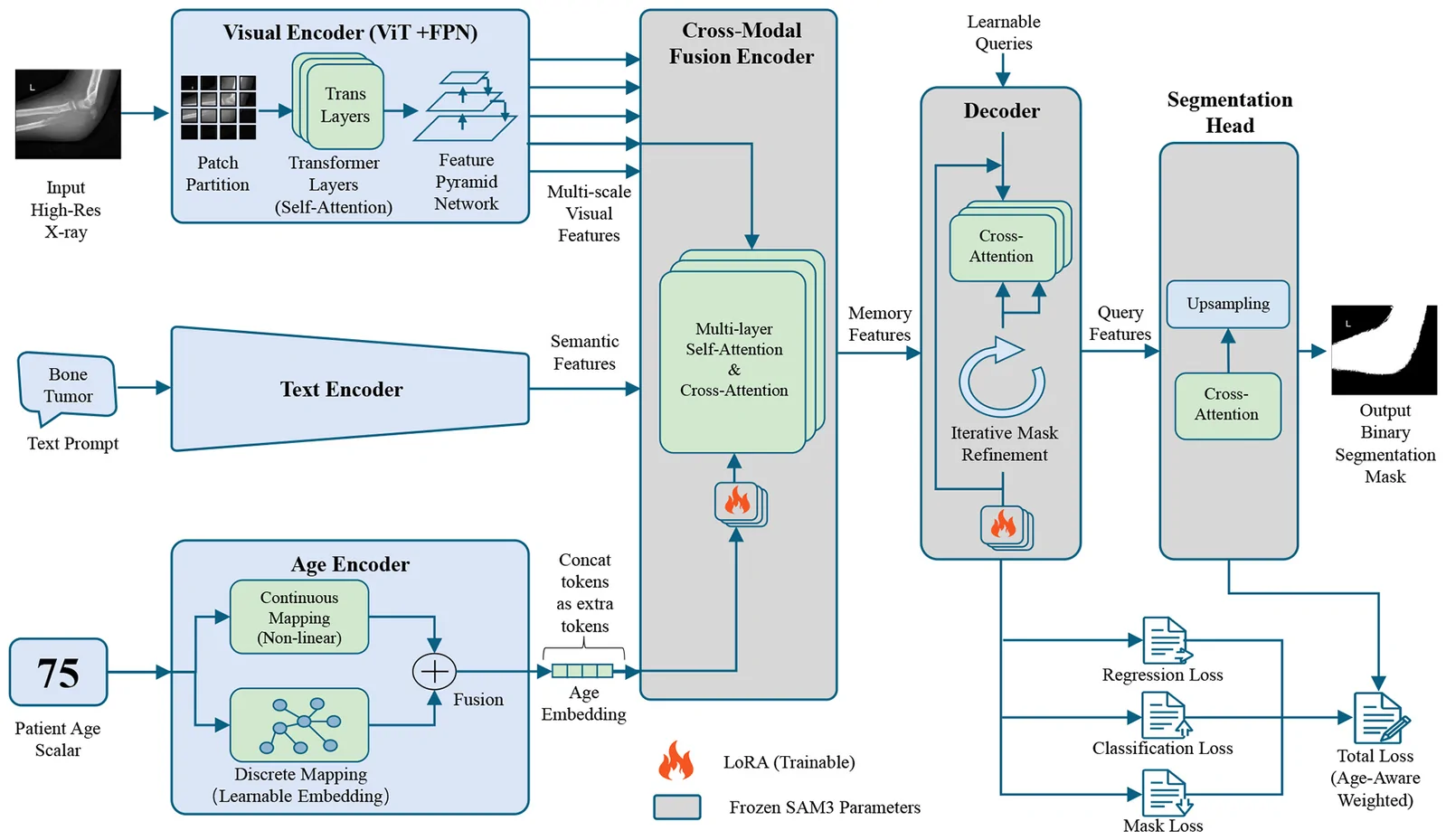
Overview of our SAM3-AgeSeg. As a novel age-aware multi-modal bone tumor segmentation framework, SAM3-AgeSeg first encodes high-resolution X-rays, text prompts, and patient age via dedicated visual, text, and age encoders. It then fuses multi-modal features through a cross-modal fusion encoder with LoRA and iterative box refinement to generate query features, and finally produces binary segmentation masks via a segmentation head.

This section provides an overview of the SAM3-AgeSeg framework, illustrated in Figure 1. SAM3-AgeSeg is an age-aware multi-modal fusion framework designed for bone tumor segmentation, tailored for elderly patients and adaptable to tumor segmentation tasks in the context of aging-related imaging variations. Unlike models that rely solely on computationally expensive attention mechanisms or pure convolutional networks, SAM3-AgeSeg integrates four core modules into a U-Net-like architecture. These four modules are multi-modal encoding, cross-modal fusion, iterative query optimization, and an age-aware segmentation head. This design preserves the model's ability to perceive age-associated imaging characteristics while maintaining computational efficiency. Structurally, the framework ensures geometrically consistent predictions of bone tumor spatial location and morphology in X-ray images through architectural constraints. Its end-to-end pipeline comprises four key stages, namely multi-branch modal encoding, physics-guided cross-modal feature fusion, iterative query optimization, and age-aware segmentation reconstruction. Initially, the high-resolution X-ray image, text prompt, and patient age scalar are projected into different modal feature spaces, capturing multi-scale visual features, and semantic features, as well as age embeddings, via the visual, text, and age encoders, respectively. Subsequently, the cross-modal fusion encoder refines multi-modal features through multi-layer self-attention and cross-attention, combined with learnable queries and iterative box optimization, to generate query features. Finally, the segmentation head generates a binary segmentation mask via upsampling and cross-attention. The model is trained using a composite loss function that integrates mask loss, box regression loss, and classification loss, adjusted with age-aware weights, to accurately locate tumor boundaries while adapting to the skeletal changes of elderly patients.

### 3.3 Multi-modal encoding with age embedding

SAM3-AgeSeg processes input information through three parallel encoding branches: the visual encoder, text encoder, and age encoder. The high-resolution bone tumor X-ray image is first fed into the visual encoder, which is based on a Vision Transformer architecture. The image is divided into fixed-size patches and mapped to a visual feature sequence that captures both global and local skeletal structural information. The text prompt is fixed as “bone tumor” for all tumor-containing images, providing semantic category information. It is encoded by the text encoder into a semantic feature vector, enabling the model to comprehend the clinical conceptual meaning of the target to be segmented. The age encoder is a distinctive module of our framework. It maps the patient age scalar to an embedding vector with the same dimensionality as the visual and textual features. The age encoder adopts a dual-path design. One path maps the normalized age value to a continuous feature representation through a non-linear multilayer perceptron. The other path maps the integer age value to a discrete feature representation through a learnable embedding table. The outputs of the two paths are summed element-wise to obtain the final age embedding. This embedding is subsequently injected into the prompt sequence of the cross-modal fusion encoder, allowing the model to perceive the patient age at the feature extraction stage and to pay particular attention to age-associated imaging features in elderly patients.

Formally, given an input image *X*, a text prompt *T*, and the patient age *a*, the outputs of the respective encoders are expressed as shown in Equations 1–3


$$\begin{array}{lll} F_{v} & = & E_{vis}\left(X\right), \end{array}$$ (1)

$$\begin{array}{lll} F_{t} & = & E_{txt}\left(T\right), \end{array}$$ (2)

$$\begin{array}{lll} F_{a} & = & E_{age}\left(a\right)=\text{MLP}\left(\frac{a}{A_{max}}\times 2-1\right)+\text{Embedding}\left(\left\lfloor a\right\rfloor \right), \end{array}$$ (3)

where $E_{vis}$, $E_{txt}$ and $E_{age}$ denote the visual encoder, text encoder and age encoder respectively, *A*_*max*_ is the maximum possible age constant in the dataset, *F*_*v*_ is the visual feature sequence, *F*_*t*_ is the semantic feature vector and *F*_*a*_ is the age embedding vector. All features are aligned and passed to the subsequent cross-modal fusion module.

### 3.4 Cross-modal fusion encoder with LoRA adaptation

The cross-modal fusion encoder receives the visual features, textual features, and the age embedding, and performs deep interaction among the four modalities through multi-layer self-attention and cross-attention mechanisms. The age embedding is concatenated to the input prompt sequence as an additional token, enabling self-attention to dynamically adjust attention weights across different visual regions based on patient age. This increases sensitivity to tumor boundaries in elderly patients. To efficiently adapt the pre-trained SAM3 weights to the bone tumor domain, especially for elderly patients, this framework uses Low-Rank Adaptation. Only low-rank matrices are injected into the attention projection layers and feed-forward network layers of the Transformer encoder and decoder, while all other parameters remain frozen. Let the original weight matrix be $W\in {\mathbb{R}}^{d_{out}\times d_{in}}$. We introduce a low-rank decomposition *W*_*lora*_ = *BA* where $B\in {\mathbb{R}}^{d_{out}\times r}$, $A\in {\mathbb{R}}^{r\times d_{in}}$, and the rank *r* is much smaller than min(*d*_*in*_, *d*_*out*_). The forward pass is then modified as shown in Equation 4


$$\begin{array}{l} h=Wx+\frac{\alpha }{r}BAx, \end{array}$$ (4)

where α is a scaling coefficient controlling the magnitude of the LoRA contribution, and *x* is the input feature. For the age-modulated variant, the LoRA output is additionally multiplied by a scalar produced by a gating network taking the age embedding as input as shown in Equations 5 and 6


$$\begin{array}{l} h=Wx+\lambda \left(a\right)\cdot \frac{\alpha }{r}BAx, \end{array}$$ (5)

$$\begin{array}{l} \lambda \left(a\right)=\sigma (w\cdot \left(a-\tau \right)) \end{array}$$ (6)

where σ denotes the sigmoid activation function, and λ(*a*) approaches 1 for older patients to strengthen the LoRA adaptation effect and approaches 0 for younger patients to preserve the original general representation of SAM3.

### 3.5 Segmentation head and age-aware loss function

The segmentation head takes the query features output by the decoder and generates a binary tumor segmentation mask Ŷ with the same spatial resolution as the input image. This is achieved through a pixel decoder that progressively upsamples features and performs cross-attention with multi-scale encoder features. The model is trained using a composite loss function comprising three components: mask loss, bounding box regression loss, and classification loss. The mask loss combines binary cross-entropy and the Dice coefficient loss to address extreme foreground-background imbalance and boundary ambiguity. The bounding box regression loss penalizes deviations between the predicted and ground-truth bounding boxes. The classification loss measures the confidence of whether a query corresponds to an existing target. To enhance segmentation accuracy for elderly patients, an age-aware loss weighting mechanism is introduced. Denote the patient age of the *i*-th sample as *a*_*i*_, and the base segmentation loss as $L_{seg}^{\left(i\right)}$. The age-weighted total loss is defined as shown in Equation 7


$$\begin{array}{l} L_{total}^{\left(i\right)}=\{\begin{array}{cc} \omega \cdot L_{seg}^{\left(i\right)}, & a_{i}>a_{th}\\ L_{seg}^{\left(i\right)}, & a_{i}\leq a_{th} \end{array} \end{array}$$ (7)

where *a*_*th*_ is the elderly threshold typically set to 60 years; the threshold of 60 years for defining the elderly population is adopted following the World Report on Aging and Health published by the World Health Organization (30), which identifies 60 years as the typical chronological age demarcating older adulthood. ω>1 is a hyperparameter controlling the loss weight for elderly samples. This design forces the model to pay more attention to gradient signals from elderly samples during backpropagation, mitigating underfitting caused by the scarcity and lower image quality of elderly cases and improving the segmentation robustness of the model in the aging population.

## 4 Experiments

In this section, we comprehensively evaluate the performance of SAM3-AgeSeg, covering both metric results and visualization results.

### 4.1 Experimental settings

The bone tumor X-ray images used in this study are obtained from the BTXRD dataset (14), which is a multi-center open-access dataset for primary bone tumors. The dataset consists of 3,746 X-ray images, including 1,879 normal bone images, 1,525 benign bone tumor images, and 342 malignant bone tumor images. All tumor images are annotated with bounding boxes and pixel-level segmentation masks, and are accompanied by clinical metadata such as patient age, gender, anatomical site, and benign-malignant and subtype labels. The data are collected from three clinical centers.

All experiments were conducted on an NVIDIA RTX PRO 6000D GPU using PyTorch. We used the AdamW optimizer with an initial learning rate of 5 × 10^−5^, weight decay of 0.01, and β = (0.9, 0.999). The batch size was set to 4 with gradient accumulation steps of 8, resulting in an effective batch size of 32. The model was trained for 100 epochs with a cosine-annealed learning-rate scheduler and 200 warmup steps. The LoRA rank *r* was set to 16, the scaling coefficient α was set to 32, and the dropout rate was 0.1. The age encoder was enabled with a dual-path design: a continuous path using a 2-layer MLP with hidden dimension 256 and ReLU activation, and a discrete path using an embedding table of size 101 with embedding dimension 256.

To ensure that all data splits contain sufficient elderly samples while rigorously preventing data leakage, we adopted a patient-level age-stratified partitioning strategy. All images were first grouped by unique patient ID to guarantee that multiple radiographic views originating from the same patient or lesion were confined to a single subset. The patients were then divided into two strata: the elderly group (age ≥ 60) and the non-elderly group (age < 60). Within each stratum, we randomly allocated 70% of patients to the training set, 10% to the validation set, and 20% to the test set. This patient-level splitting eliminates the risk of images being correlated across different subsets, ensuring an unbiased evaluation of model performance. The final training, validation, and test sets were then formed by combining the corresponding subsets from both strata. This strategy ensures that the proportion of elderly patients in each split consistent with that of the entire dataset, thereby enabling a fair and comprehensive evaluation of the model's adaptability to the aging population. Table 1 presents the detailed statistics of the resulting dataset partitions.

**Table 1:** Dataset statistics after age-stratified random partitioning.

Split	Total images	Elderly(≥60)	Non-elderly(< 60)	Elderly proportion
Training set	2,618	365	2,240	13.94%
Validation set	377	53	324	14.02%
Test set	751	109	655	14.51%
Total	3,746	527	3,219	14.07%

This study uses two widely used metrics in medical image segmentation to quantitatively evaluate the model performance. The Dice Similarity Coefficient (Dice) measures the overlap between the predicted segmentation and the ground truth, reflecting their spatial consistency. The Intersection over Union (IoU) measures the ratio of the intersection area to the union area between the predicted and ground truth regions, assessing the accuracy of region localization. Both metrics range from 0 to 1, with higher values indicating better segmentation performance. The mathematical formulas are presented below as shown in Equations 8 and 9


$$\begin{array}{lll} \text{Dice} & = & \frac{2\times |P\cap G|}{\left|P|+|G\right|} \end{array}$$ (8)

$$\begin{array}{lll} \text{IoU} & = & \frac{\left|P\cap G\right|}{\left|P\cup G\right|} \end{array}$$ (9)

where *P* denotes the set of pixels predicted as positive (tumor region); *G* denotes the set of ground truth positive pixels; |*P*∩*G*| represents the number of pixels in the intersection of the predicted and ground truth regions; |*P*| and |*G*| represent the number of pixels in the predicted and ground truth regions, respectively; |*P*∪*G*| represents the number of pixels in the union of the two regions.

### 4.2 Comparison with state-of-the-art methods

To comprehensively evaluate the performance of the SAM3-AgeSeg model, we compare it with several state-of-the-art methods, including U-Net (16), Attention U-Net (17), TransUNet (19), Swin-UNet (18), SAM (23), MedSAM (24), SAMed (31) and MedSAM3 (28). All baseline methods were trained or fine-tuned on the BTXRD dataset using the same training, validation, and test split as SAM3-AgeSeg. U-Net, Attention U-Net, TransUNet, and Swin-UNet were trained from scratch on the BTXRD dataset. SAM, MedSAM, SAMed, and MedSAM3 were initialized with their official pre-trained weights and fine-tuned under the same text-only prompt setting. All results are averaged over five repeated experiments. The experimental results are shown in Table 2, note that all segmentation metrics are computed exclusively on tumor-positive images; normal images without tumor masks are excluded from the calculation.

**Table 2:** Model performance on the the validation / test set.

	Metric (validation set)				Metric (test set)			
Model	Dice(all)	IoU(all)	Dice(elderly)	IoU(elderly)	Dice(all)	IoU(all)	Dice(elderly)	IoU(elderly)
U-Net (16)	0.7832	0.7241	0.7413	0.6805	0.7914	0.7332	0.7506	0.6898
Attention U-Net (17)	0.8325	0.7387	0.7851	0.7103	0.8422	0.7984	0.7930	0.7185
TransUNet (19)	0.8553	0.8101	0.8087	0.7325	0.8655	0.8209	0.8163	0.7404
Swin-UNet (18)	0.8685	0.8237	0.8252	0.7480	0.8783	0.8352	0.8245	0.7568
SAM (23)	0.8387	0.7952	0.7924	0.7163	0.8482	0.8007	0.7901	0.7235
MedSAM (24)	0.8691	0.8283	0.8365	0.7544	0.8799	0.8339	0.8236	0.7596
SAMed (31)	0.8847	0.8372	0.8467	0.7705	0.8901	0.8503	0.8567	0.7724
MedSAM3 (28)	0.8902	0.8453	0.8521	0.7782	0.8974	0.8564	0.8612	0.7773
SAM3-AgeSeg	**0.8959**	**0.8489**	**0.8736**	**0.7785**	**0.8998**	**0.8607**	**0.8849**	**0.8024**

Bold values indicate the best performance in each column.

As shown in the table, SAM3-AgeSeg consistently outperforms all compared methods across both Dice and IoU metrics on the validation and test sets. The superior performance of SAM3-AgeSeg can be attributed to several key factors. First, by leveraging the powerful visual foundation model SAM3 as the backbone, our model inherits robust feature extraction and generalization capabilities that traditional CNN-based or hybrid architectures lack. Second, the introduction of the age-embedding module enables the model to explicitly incorporate patient age information into the segmentation process, allowing it to dynamically adapt to age-specific imaging characteristics such as the blurred tumor boundaries commonly observed in elderly patients. Third, the use of LoRA for efficient fine-tuning preserves the pre-trained knowledge of SAM3 while effectively adapting it to the specialized domain of bone tumor segmentation, thereby avoiding the catastrophic forgetting problem that often plagues full fine-tuning approaches. Fourth, the age-aware loss weighting mechanism further enhances the model's focus on challenging elderly samples, improving boundary delineation where conventional methods tend to fail.

Specifically, on the validation set, SAM3-AgeSeg achieves the highest Dice and IoU scores, demonstrating superior segmentation accuracy and spatial overlap with ground truth annotations. On the test set, SAM3-AgeSeg maintains its leading position, significantly outperforming both generic segmentation models (U-Net, Attention U-Net) and transformer-based architectures (TransUNet, Swin-UNet), as well as SAM-based variants (MedSAM, SAMed, MedSAM3). Notably, even compared to MedSAM, SAMed, and MedSAM3, which are specifically designed for medical image segmentation, our model shows clear advantages, particularly on elderly patient samples where age-related imaging variations pose significant challenges. In summary, the comparative experiments with a diverse set of mainstream and advanced models fully validate the effectiveness and superiority of SAM3-AgeSeg for bone tumor segmentation in the aging population.

### 4.3 Results visualization

**Figure 2:**
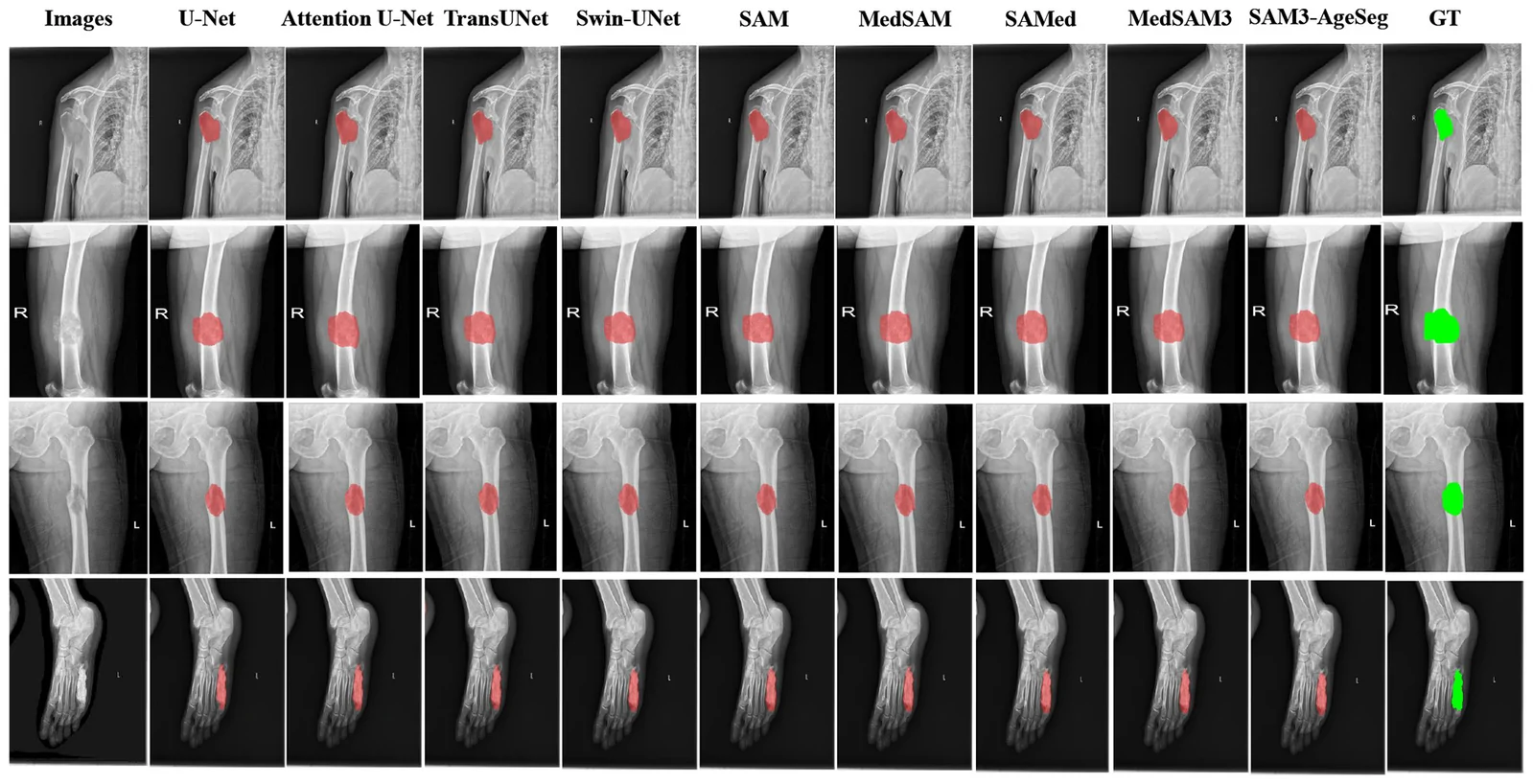
Comparative visualization of bone tumor segmentation results across different models and ground truth (GT).

To qualitatively evaluate the segmentation performance of SAM3-AgeSeg and compare it with other state-of-the-art methods, we visualized the segmentation results on representative test samples from the BTXRD dataset. Figure 2 presents a comparative visualization of bone tumor segmentation results across different models, including U-Net, Attention U-Net, TransUNet, Swin-UNet, SAM, MedSAM, SAMed, MedSAM3, and our proposed SAM3-AgeSeg, alongside the ground truth (GT) annotations.

Traditional CNN-based models such as U-Net and Attention U-Net produce coarse and incomplete segmentation masks, often failing to capture precise tumor boundaries, particularly when tumors are adjacent to normal bone tissue. Transformer-based models such as TransUNet and Swin-UNet demonstrate improved boundary delineation but still suffer from under-segmentation or over-segmentation in challenging regions.

Among the SAM-based variants, MedSAM and SAMed achieve more complete tumor coverage than vanilla SAM, benefiting from medical-domain fine-tuning. However, they still struggle with accurately segmenting tumor boundaries in elderly patients. MedSAM3, as the strongest baseline, produces relatively accurate segmentation results but occasionally misses subtle tumor extensions or includes false-positive regions near the degenerated bone.

In contrast, SAM3-AgeSeg consistently generates segmentation masks that are closest to the ground truth annotations across all displayed cases. The predicted masks exhibit clearer and more complete tumor boundaries, especially in challenging scenarios involving elderly patients with blurred lesion margins. This qualitative observation aligns with the quantitative results reported in Table 2, further validating the effectiveness of our age-adaptive design in addressing the imaging challenges associated with aging.

### 4.4 Ablation studies

To systematically assess the contribution of each key component in SAM3-AgeSeg, we conducted a comprehensive ablation study on the BTXRD dataset. Model A serves as the SAM3 baseline without age conditioning: vanilla SAM3 with text-only prompting, no LoRA, no age embedding, and no age-aware loss. Models B–H incrementally add LoRA, age embedding, and age-aware loss weighting. The comparison between Model A and Model H directly quantifies the overall gain brought by our age-aware modifications, as shown in Table 3. The performance, measured by Dice and IoU on both validation and test sets, clearly reveals the effectiveness and interaction of the age embedding module, LoRA fine-tuning, and age-aware loss weighting. To ensure rigorous evaluation and avoid test-set leakage, all hyperparameter tuning and ablation analyses were conducted exclusively on the validation set. The loss weight ω, LoRA rank *r*, and other hyperparameters were selected based on validation performance. The age threshold of 60 years was adopted following the World Health Organization's *World Report on Aging and Health* rather than tuned as a hyperparameter. The eight ablation variants (Models A–H) were first evaluated on the validation set to assess the contribution of each component. After confirming the optimal configuration, the final model was evaluated on the test set only once.

**Table 3:** Ablation studies of age embedding, LoRA, and age-aware loss weighting on the BTXRD dataset.

Models	Age embedding	LoRA	Age-aware loss	Test dice(all)	Test Iou(all)	Test dice(elderly)	Test Iou(elderly)
A	×	×	×	0.8508	0.8055	0.7929	0.7264
B	✓	×	×	0.8648	0.8186	0.8334	0.7719
C	×	✓	×	0.8549	0.8087	0.8217	0.7563
D	×	×	✓	0.8528	0.7964	0.8284	0.7657
E	✓	✓	×	0.8797	0.8308	0.8538	0.7891
F	×	✓	✓	0.8835	0.8394	0.8507	0.8006
G	✓	×	✓	0.8701	0.8217	0.8432	0.7811
H	✓	✓	✓	**0.8998**	**0.8607**	**0.8849**	**0.8024**

Bold values indicate the best performance in each column.

### 4.5 Effectiveness of age embedding

Introducing the age embedding module (Model B) yields a substantial performance improvement over the baseline (Model A). The Dice and IoU are markedly improved, particularly on the elderly subset. This decisive gain underscores the paramount importance of incorporating age information for bone tumor segmentation in the aging population.

### 4.6 Effectiveness of LoRA fine-tuning

Incorporating LoRA fine-tuning (Model C) also leads to a clear, albeit more moderate, improvement compared to the baseline. This improvement is crucial for enhancing the model's ability to adapt the SAM3 foundation model to the bone tumor domain while preserving its general visual priors.

### 4.7 Effectiveness of age-aware loss weighting

Using the age-aware loss weighting (Model D) provides a consistent performance benefit, validating its design tailored for the elderly population. The adaptive weighting mechanism forces the model to focus on challenging elderly samples during training.

### 4.8 Synergistic effects and final integration

Ablation results reveal clear synergistic effects among the modules. Any two-module combination outperforms its individual counterparts, demonstrating their complementary roles. The full model, integrating all three components, achieves the best overall performance and robustly demonstrates that each module is indispensable. This co-design enables SAM3-AgeSeg to optimally balance segmentation accuracy and robustness for the aging population.

## 5 Conclusion

In this paper, we proposed SAM3-AgeSeg, an adaptive segmentation model for bone tumors in the aging population. By integrating age embedding, LoRA-based fine-tuning, and age-aware loss weighting within the SAM3 framework, our model effectively addresses blurred tumor boundaries commonly observed in elderly patients. Experiments on the BTXRD dataset demonstrate that SAM3-AgeSeg outperforms existing methods on both overall and elderly-specific subsets. Future work includes extending the framework to other aging-affected medical imaging tasks and exploring adaptive weighting strategies to further improve generalizability.

## Data Availability

The original contributions presented in the study are included in the article/supplementary material, further inquiries can be directed to the corresponding authors.
